# Current status and future challenges of genotoxicity OECD Test Guidelines for nanomaterials: a workshop report

**DOI:** 10.1093/mutage/gead017

**Published:** 2023-05-26

**Authors:** Shareen H Doak, Cristina Andreoli, Michael J Burgum, Qasim Chaudhry, Eric A J Bleeker, Cecilia Bossa, Josefa Domenech, Damjana Drobne, Valérie Fessard, Nina Jeliazkova, Eleonora Longhin, Elise Rundén-Pran, Maciej Stępnik, Naouale El Yamani, Julia Catalán, Maria Dusinska

**Affiliations:** Institute of Life Science, Swansea University Medical School, Singelton Park, Swansea, SA2 8PP Wales, United Kingdom; Department of Environment and Health, Istituto Superiore di Sanità, Rome, Italy; Institute of Life Science, Swansea University Medical School, Singelton Park, Swansea, SA2 8PP Wales, United Kingdom; University of Chester, Parkgate Road, Chester, United Kingdom; National Institute for Public Health and the Environment (RIVM), PO Box 1, 3720 BA Bilthoven, The Netherlands; Department of Environment and Health, Istituto Superiore di Sanità, Rome, Italy; Finnish Institute of Occupational Health, Box 40, Työterveyslaitos, 00032 Helsinki, Finland; University of Ljubljana, Biotechnical Faculty, Department of Biology, Vecan pot 111, 1000 Ljubljana, Slovenia; ANSES French Agency for Food, Environmental and Occupational Health and Safety, Fougères Laboratory, Toxicology of Contaminants Unit, 10b rue Claude Bourgelat, Fougères 35306, France; Ideaconsult Ltd., Sofia, Bulgaria; NILU-Norwegian Institute for Air Research, Instituttveien 18, Kjeller 2002, Norway; NILU-Norwegian Institute for Air Research, Instituttveien 18, Kjeller 2002, Norway; QSAR Lab Ltd., Trzy Lipy 3 St., Gdańsk, Poland; NILU-Norwegian Institute for Air Research, Instituttveien 18, Kjeller 2002, Norway; Finnish Institute of Occupational Health, Box 40, Työterveyslaitos, 00032 Helsinki, Finland; Department of Anatomy, Embryology, and Genetics, University of Zaragoza, 50013 Zaragoza, Spain; NILU-Norwegian Institute for Air Research, Instituttveien 18, Kjeller 2002, Norway

**Keywords:** nanomaterials, nanosafety, genotoxicity, *in vitro* 3D models, OECD, new approach methodologies (NAMs)

## Abstract

Genotoxicity testing for nanomaterials remains challenging as standard testing approaches require some adaptation, and further development of nano-specific OECD Test Guidelines (TGs) and Guidance Documents (GDs) are needed. However, the field of genotoxicology continues to progress and new approach methodologies (NAMs) are being developed that could provide relevant information on the range of mechanisms of genotoxic action that may be imparted by nanomaterials. There is a recognition of the need for implementation of new and/or adapted OECD TGs, new OECD GDs, and utilization of NAMs within a genotoxicity testing framework for nanomaterials. As such, the requirements to apply new experimental approaches and data for genotoxicity assessment of nanomaterials in a regulatory context is neither clear, nor used in practice. Thus, an international workshop with representatives from regulatory agencies, industry, government, and academic scientists was convened to discuss these issues. The expert discussion highlighted the current deficiencies that exist in standard testing approaches within exposure regimes, insufficient physicochemical characterization, lack of demonstration of cell or tissue uptake and internalization, and limitations in the coverage of genotoxic modes of action. Regarding the latter aspect, a consensus was reached on the importance of using NAMs to support the genotoxicity assessment of nanomaterials. Also highlighted was the need for close engagement between scientists and regulators to (i) provide clarity on the regulatory needs, (ii) improve the acceptance and use of NAM-generated data, and (iii) define how NAMs may be used as part of weight of evidence approaches for use in regulatory risk assessments.

## Introduction

The assessment of genotoxicity is based on both *in vitro* and *in vivo* studies for most chemicals. However, standard genotoxicity assays originally designed for conventional chemicals, have shown certain limitations when applied to nanomaterials, especially for *in vitro* testing [1]. There has therefore been the suggestion for adaptations of several validated OECD Test Guidelines (TGs) to account for nano-specific considerations, and the development of supporting Guidance Documents (GDs), several of which are currently in progress [2]. In parallel, new approach methodologies (NAMs), or non-animal approaches, are being developed that have the potential to provide relevant information on the mechanisms of genotoxic action of nanomaterials. However, the implementation of new and/or adapted OECD TGs, development of new OECD GDs, including those relating to NAMs, and the requirements to apply the existing or new data for genotoxicity assessment of nanomaterials in a regulatory context, remain challenging.

To consider, discuss and address these issues, a workshop was organized focussed on ‘*Current status and future challenges of genotoxicity test guidelines (TGs) for nanomaterials*’. This workshop was a satellite event of the final conference of the European Commission Horizon 2020 NMBP-13 projects. The conference ‘*Future-proof Approaches for Risk Governance—Lessons Learned from Nanomaterials*’, held at the OECD premises in Paris (France) on 24–25 January 2023, was organized by the Horizon 2020 projects NANORIGO (grant agreement no. 814530), RiskGONE (grant agreement no. 814425), and Gov4Nano (grant agreement no. 814401), in collaboration with the OECD’s Working Party on Manufactured Nanomaterials (WPMN). The objectives of the workshop were:

1. to present the state-of-the-art for key assays and NAMs that could, and have been applied for evaluating the genotoxicity of nanomaterials;
2. to gain insight and understanding of what is needed to implement validated assays and NAMs, and how to apply the data generated by these methods in the regulatory assessment of nanomaterials.

The workshop consisted of several formal presentations from key participants outlining current progress in the field and the remaining challenges for robust genotoxicity testing of nanomaterials. This was followed by a roundtable discussion with a group of experts involved in regulatory risk assessment of both chemicals and nanomaterials, the industry, and research groups, to consider specific points in more detail. Participants in the roundtable discussions included representatives from the European Food Safety Authority (EFSA), Scientific Committee on Consumer Safety (SCCS), Finnish Institute of Occupational Health (FIOH, Finland), the Dutch National Institute for Public Health and the Environment (RIVM, The Netherlands), Istituto Superiore di Sanità (ISS, Italy), French Agency for Food, Environmental and Occupational Health and Safety (ANSES, France), as well as individuals from industry and academia. Since the approaches or views on the application of standard genotoxicity tests for evaluating nanomaterials can vary between different organizations, risk assessment committees, and countries, it was intended that the workshop would also facilitate a move towards greater harmonization and standardization of the hazard assessment approaches required.

## General considerations for *in vitro* genotoxicity OECD TGs

Genotoxicity testing of nanomaterials is challenging, as in most cases, their behaviour cannot be predicted solely from that of the chemicals from which they are derived. They may differ in terms of particle size, shape, and surface properties, and their high surface–volume ratio may make them more or differently reactive compared with conventional chemical forms. This reactivity means that they can interact with their environment, that may also lead to the formation of a corona (e.g. of proteins) on the particle surface, which can further modify their chemical behaviour. Nanoparticles also tend to aggregate and agglomerate more than larger sized particles. Therefore, characterizing nanomaterials in the pristine state and after dispersing in the exposure medium is crucially important, to enable correlating physicochemical features of the test material with the toxicological responses [3, 4]; although the question of dosimetry demands further research and harmonization [5].

Like chemicals, some nanomaterials may cause genotoxicity in different ways: i.e. through primary direct interaction with the DNA molecule; primary indirect mechanisms via, e.g. oxidative stress (e.g. production of reactive oxygen species), or interaction of nanomaterials with proteins involved in DNA replication; or through secondary genotoxicity, mediated via an inflammatory response of macrophages and neutrophils [6, 7]. The standard test guidelines for gene mutation (TG476) and clastogenicity/aneugenicity (TG487, TG473) used to assess genotoxicity of chemicals in mammalian cells may not be fully applicable to nanomaterials and therefore could require modification [1, 3, 8]. Whilst other tests without OECD TGs, such as the *in vitro* comet assay, can provide useful information when assessing the potential genotoxicity and modes of action of nanomaterials [9]. In addition, for nanomaterials, it is important to evaluate and demonstrate cell exposure, that can be assessed by cellular uptake and intracellular distribution, to appropriately interpret negative genotoxicity test results [1, 10].

Given, the current shortcomings in the genotoxicity testing framework, the objective of the RiskGONE project (grant agreement no. 814425) was to contribute to the standardization and validation of Standard Operation Procedures (SOPs) for assessing nanomaterials. RiskGONE operated through interlaboratory studies (‘round robin’ exercises) by testing the same nanomaterials and using a common approach for their dispersion and characterization. The work has been completed, and results have been made available publicly in the form of project deliverables, papers, and training materials. Furthermore, SOPs have been validated, and an SOP for the *in vitro* comet assay and colony forming ability will be proposed for inclusion in the OECD Test Guideline Programme for development of new Test Guidelines [11–14]. Additionally, the interference of nanomaterials with test components and/or damage detection systems of *in vitro* assays has been an issue. Through RiskGONE, recommendations have been drawn to ensure that interference controls are always included in genotoxicity testing approaches to avoid misleading positive/negative results [15].

In summary, important considerations when working with nanomaterials include the need for the minimum requirements for characterization to be met, provision of a clear description of sample preparation (including dispersion), evaluation of dosimetry, demonstration of cellular uptake, optimizing exposure time and concentrations to be tested, and the inclusion of interference controls, in addition to the positive, negative, and solvent controls. Incorporation of these aspect in an OECD TG or GD advising on a general approach to the testing of nanomaterials, would be very useful for their future application in hazard and risk assessment.

## Genotoxicity testing with NAMs for regulatory purposes

It has long been recognized that standard *in vitro* genotoxicity testing approaches have limitations for nanomaterials [1, 8]. For example, an OECD expert panel concluded in 2013 on the need to adapt the *in vitro* mammalian cell micronucleus test (OECD TG487) to facilitate evaluation of insoluble materials (including nanomaterials), which are excluded from its applicability domain [16]. In response to this issue, an OECD project was initiated to develop a new OECD GD on adaptation of the *in vitro* micronucleus assay (OECD TG487) for testing of manufactured nanomaterials. The GD was published in September 2022 [17].

Whilst the ongoing efforts have focussed on adapting existing OECD TGs to ensure that they are applicable for nanomaterials, limitations remain as existing *in vitro* approaches lack physiological relevance, often do not enable evaluation of long-term exposure effects that may be crucial for nanomaterials, and do not always inform on the wide spectrum of mechanisms of action underpinning genotoxicity, and the potential for subsequent carcinogenicity. However, NAMs can provide the opportunity to overcome these issues and there have been substantial developments in this area over recent years [7, 18–20]. The 3D reconstructed skin micronucleus (RSMN) assay is one such example, which has been validated for chemicals and demonstrated as a suitable test for dermally applied compounds. This NAM has also recently been accepted into the OECD Work Plan for the Test Guidelines Programme (Project 4.139) [21, 22]. The 3D RSMN assay has been readily applied to the evaluation of nanomaterials, where the only note of caution is in relation to the top dose applied as exposures at concentrations that are unrealistically high can result in suffocation of the model by entirely masking the surface [22, 23].

Standard genotoxicity testing approaches typically only evaluate primary genotoxicity (direct and/or indirect DNA damage mechanisms), but do not efficiently report on secondary genotoxicity, which is detected *in vivo* as a consequence of inflammation [7]. This is particularly problematic for nanomaterials, which can readily induce inflammation, e.g. following inhalation, and in some cases have the capacity to induce secondary genotoxic effects *in vivo* [24, 25]. The development of NAMs based on the co-culture of both immune and lung epithelial cells has however addressed this issue, enabling the detection of secondary genotoxic mechanisms *in vitro*, thereby improving the scope of identification of *in vivo* relevant mechanisms detected by the *in vitro* systems [26, 27].

Whilst dermal and inhalation exposure routes are considered predominant for nanomaterials, other organs of concern include the gastrointestinal tract (GIT) following oral exposure, and the liver, which is a key organ in which nanomaterials are known to accumulate and remain for long periods of time if they traverse these biological barriers. More advanced culture systems representing both the GIT and liver have been developed for genotoxicity testing of nanomaterials [18]. For example, a triple co-culture model representing both a healthy and inflamed GIT have been designed to support DNA damage testing of nanomaterials using the comet assay [28]. Whilst not yet applied to the evaluation of nanomaterials, the reconstructed intestine micronucleus cytome (RICyt) assay could be a promising tool for their genotoxicity testing in the future [29]. With respect to the liver, a 3D spheroid model based on the HepG2 liver epithelial cell line has been applied to evaluate nanomaterial genotoxicity and is a system that can report on a variety of endpoints including genotoxicity (micronucleus and comet assay), cytotoxicity, metabolic activity, gene expression/transcriptomics analysis, oxidative stress, and inflammatory response [30–34]. The SOPs designed for this NAM have undergone a preliminary interlaboratory trial, demonstrating that the system was reproducible and could be readily transferred to other laboratories [35]. Additionally, a 3D HepaRG liver spheroid system has been developed and optimized for the comet assay and is now being utilized to evaluate nanomaterials through the RiskGONE project [36].

The broad field of NAMs to better support regulatory risk decision-making without the use of laboratory animals is clearly gaining momentum, with a variety of new and promising methodologies emerging. These novel approaches not only have the capacity to minimize the need for *in vivo* testing but also enable better evaluation of the mechanisms associated with nanomaterial-induced genotoxicity. The future challenge, however, is the speed at which these NAMs may be integrated into a decision-making framework for regulatory risk assessments. The validation of a new method can take well over a decade, and it is crucial to move forward in the meantime to establish the use of those NAMs that may not have been formally validated but can be accepted as being scientifically valid as part of weight of evidence (WoE) for regulatory risk assessments.

## The *in vivo* micronucleus assay

From a regulatory perspective, the assessment of the mutagenicity of chemicals relies on a battery of standard *in vitro* assays that, according to the regulations and if giving a positive result, should be confirmed, or discounted by follow-up *in vivo* assays [37–39]. In the case of nanomaterials, a similar approach is also recommended within the genotoxicity testing roadmap [1].

The mammalian erythrocyte micronucleus assay is a validated test that allows the detection of damage induced to the chromosomes or the mitotic apparatus of bone marrow erythroblasts [40]. It is usually preferred to the Mammalian Bone Marrow Chromosomal Aberration test because it is less time-consuming, and it does not require as high a level of expertise for the analyses [41]. In addition, it can detect both clastogenic and aneugenic effects. However, there are several concerns about its applicability for the *in vivo* assessment of nanomaterials. Most of them refer to the possibility of false negative results if the material or its secondary mediators do not reach the target tissue [1, 4, 39, 42]; or false positive results due to non-specific systemic toxicity induced by high doses [4]. Regarding the former, recent recommendations from different regulatory agencies highlight the need for relevant toxicokinetic data to assess whether the nanomaterial reaches the target tissue, which is of particular importance where the target tissue is not the site of contact [9, 37, 38]. This can be more problematic for nanomaterials as they may behave differently to conventional chemicals, for which we can generally predict toxicokinetics. Thus, there is an ongoing OECD project to develop a nano-specific TG for toxicokinetics to facilitate the generation of these data in a harmonized manner (https://nanoharmony.eu/2022/10/18/report-available/).

Based on the review by Rodriguez-Garraus *et al.* on silver nanoparticles, the outcomes of the *in vivo* mammalian erythrocyte micronucleus assay were not affected by the route of exposure or treatment schedule [43]. This result was reported despite other authors previously noting an association between positive results with nanomaterials and repeated administrations [44]. Instead, this was an effect resulting from surface functionalization, with uncoated and citrate-Ag nanoparticles producing positive results. This may have been due to the material functionalization affecting the rate of dissolution and, consequently, the possibility of silver ions reaching the bone marrow and exerting an effect. However, none of the studies considered in this review were conducted through the respiratory exposure route, which is one of the most relevant regarding nanomaterial exposure [38]. When focussing on the respiratory route, a more recent review found that positive outcomes were reported with TiO_2_ nanoparticles, whereas nanofibres (mainly carbon nanotubes) recorded negative results [45]. On the other hand, Horibata *et al.* focussed on genotoxicity studies performed with Mitsui-7, the only multiwalled carbon nanotube that has been classified as a possible carcinogen to humans (Group 2B) [46, 47]. They observed that any assay assessing genotoxic effects of Mitsui-7 in the bone marrow or blood erythrocytes generated negative outcomes. This could of course be because Mitsui-7 may not induce chromosome damage. However, the same authors reported a significant increase in the frequency of micronuclei in the lung tissue 24 h after a single intratracheal instillation of mice with 0.5 mg/animal of Mitsui-7, whereas no significant induction of micronuclei was detected in the erythrocytes of the same animals. Similar results had been previously observed by Catalán *et al.* [44] when analysing the frequency of micronuclei in the lung and erythrocytes of mice 24 h after an inhalation exposure to Mitsui-7 (10.8 mg/m^3^, 4 days, 4 h/day) [44]. Furthermore, whereas Horibata *et al.* [46] used a soluble chemical compound (ethyl methanesulfonate) as a positive control that was able to induce micronuclei in both types of tissues, the particulate material used by Catalán *et al*. [44]—tungsten cobalt mixture—only induced micronuclei in the lung tissue. This emphasizes the fact that some materials may not be able to cross the air–blood barrier into the circulation or, even where it does happen, the current time schedules used in the *in vivo* micronucleus assay may not be sufficient for all nanomaterials to reach the systemic target tissue.

In summary, the current administration and sampling times recommended by the OECD TG 474 may not be suitable for all nanomaterials. Following the 3Rs’ principles, it would be advised to couple this assay with the toxicokinetics studies already required by the regulatory guidelines or with other *in vivo* assays, i.e. chronic inhalation studies that have been adapted for nanomaterials [OECD TGs 412 (28-day) and 413 (90-day), respectively] [48, 49]. Furthermore, the assessment of micronuclei at the site of contact (e.g. lung tissue), and the possibility of substituting *in vivo* studies by *in vitro* NAMs should also be considered in the near future.

## Reusability of data: general principles

The New European Bauhaus is a key component of the European Green Deal. It is focussed on promoting the reuse of resources and the integration of diverse knowledge and experiences through collaborative cross-disciplinary approaches, multistakeholder engagement, and communication. These approaches prioritize the integration of diverse perspectives and expertise, which can lead to more effective solutions and more sustainable outcomes [50]. Multistakeholder engagement is also important in these initiatives as it enables different groups to work together towards a common goal and ensures that diverse voices are heard and considered. In recent years, the rise of open data initiatives and open-source software has made it easier for individuals and organizations to share and reuse knowledge and data [51].

### Data reuse

This refers to the practice of using existing data for new purposes, rather than collecting new data. This approach can help to increase resource efficiency, reduce costs, save time, and improve the quality of decision-making by providing more comprehensive and accurate information. The reuse of resources is a critical aspect of the circular economy and can contribute significantly to the success of the European Green Deal.

### Harmonization of data reporting

Currently, the implementation of data sharing policies, activities, and norms is not consistent across knowledge domains. Different disciplines have varying practices regarding data sharing and terminology use, which can make it difficult to compare and integrate data from different sources. To address these challenges, there have been efforts to develop common data sharing standards and practices across disciplines and publication venues. For example, the FAIR (Findable, Accessible, Interoperable, and Reusable) data principles provide a framework for ensuring that data are shared in a way i.e. easily discoverable, accessible, and reusable [52, 53]. To support this initiative, data reporting templates are being established with a purpose of setting *common standards and practices for data sharing and description*, so researchers can promote more efficient and effective *data sharing and reuse* across different domains and disciplines.

### Data management plan

The data management plan (DMP) is a useful tool to guide data and knowledge sharing and integration of knowledge. A DMP is a document that outlines how research data will be managed throughout its lifecycle, including how it will be collected, documented, stored, shared, and preserved. By providing a structured approach to data management, DMPs can help to ensure that data are effectively managed and shared in a way that supports collaboration and knowledge integration. By following the guidelines outlined in a DMP, researchers can ensure that their data are well documented and preserved, which can enable others to build on their work, increase the impact of their research, and promote collaboration and innovation. DMPs can also support the integration of knowledge from different disciplines and stakeholders, which can lead to more effective solutions and more sustainable outcomes.

One of the challenges in data management is ensuring that data are properly reported and documented in a way that enables its reuse. A solution to this challenge is the development and application of harmonized data reporting templates, which can help to harmonize and streamline data reporting. Efforts to develop data reporting templates are ongoing in various projects. By using standardized templates and approaches, researchers can make their data more easily discoverable, accessible, interoperable, and reusable (FAIR) [54]. A combination of standardized practices and advanced technologies can help to build a more robust and efficient data management ecosystem that facilitates data sharing and integration, ultimately leading to more impactful and sustainable research outcomes [50, 55].

Data entry templates for several genotoxicity assays [comet assay, HPRT gene mutation assay, acute exposure 2D/3D cytokinesis blocked micronucleus (CBMN) assay, and long-term exposure 3D CBMN assay] as well as cytotoxicity assays have been designed with active participation of the nanosafety and genotoxicity communities, and data providers, and are available through Nano Safety Data Interface Template Wizard [54]: https://search.data.enanomapper.net/datatemplate. New templates can be added, and existing templates modified, if necessary. The templates are aligned with existing OECD TGs (where appropriate) and organized as easy-to-use Excel files. These templates are the entry point to a FAIRification workflow and conversion to machine readable formats, thus are considered FAIR enabling resources [56]. Harmonizing data reporting therefore allows improved comparison of results from different laboratories, and eases its wider use by scientists, industry, risk assessors, or modellers.

## Outcomes from the roundtable discussion

Following the presentations there was an open discussion session led by a roundtable of representatives from regulatory bodies, government risk organizations and industry aimed at discussing the current key challenges in nanomaterials regulatory genetic toxicology. The outcomes of these discussions are summarized below:

1. Do our approaches for nanomaterial genotoxicity testing identify those that could cause cancer and/or germ cell mutagenicity; do reference data on genotoxic nanomaterials leading to carcinogenicity exist?

This question presents a challenge for nanomaterials due to the limited available evidence for carcinogenicity across the broad classes of nanomaterials. The current OECD TGs and standard genotoxicity testing approaches are typically based on short-term exposures, but cancer development is a long-term process. There is significant evidence to demonstrate that some nanomaterials have the potential to accumulate in various organs of the body; thus, long-term exposure is likely to be more relevant for evaluating genotoxicity, particularly those associated with systemic tissues. It is also important to note that whilst genotoxicity is a surrogate measure for possible carcinogenicity, the latter is a multistage process and consequently, its assessment requires a battery of assays in a tiered approach. Moreover, carcinogenicity can occur without genotoxic events, affecting various intracellular processes that are not detected by the *in vitro* OECD TGs testing battery; these compounds are non-genotoxic carcinogens [57]. Nanomaterials could also have the potential to lead to cancer through such mechanisms. Whatever the mechanism, it can be captured in Adverse Outcome Pathways (AOPs), where cancer is the Adverse Outcome (AO). Whist not all assays within a battery may need to be performed, it is considered that key endpoints [e.g. linked to Key Events (KEs) within an AOP] should be recognized and addressed. For instance, an increased production of inflammatory cytokines, accomplished by increased formation of reactive oxygen species that could result in higher rates of DNA damage and mutations have been described as KEs in the development pathways of lung (e.g. AOPs 303, 416, 417, and 451) and breast (e.g. AOPs 439) cancer. Increased production of reactive oxygen species and reactive nitrogen species can be molecular initiating events leading to mutations (e.g. AOP 296) and induction of breast cancer (e.g. AOP 294); or, when release of these species is chronic, is associated with gastric cancer (e.g. AOP 298) (aopwiki.org). Currently, methodologies to cover the full spectrum of molecular and/or cellular endpoints defined within carcinogenicity AOPs are lacking. However, this issue is not restricted to nanomaterials and is also applicable to soluble chemicals.

Novel approaches based on mechanisms of action therefore require further development to ensure the ability to detect both genotoxic and non-genotoxic carcinogens is available [57]. Nonetheless, it was also recognized that a significant barrier in the development of robust genotoxicity testing approaches for nanomaterials was the current lack of appropriate positive nano-sized controls and reliable reference nanomaterials.

2. What are the major gaps in the testing strategy for genotoxicity assessment of nanomaterials? How can they be filled?

It was considered by the roundtable group that, in general, technical issues, not necessarily gaps, exist with specific OECD TGs for genotoxicity testing. Furthermore, how these TGs can be/are implemented in practice is problematic because of omissions in nano-specific data requirements. For example, often adequate nanomaterial physicochemical characterization data are not provided; in which case a regulatory evaluator is not able to correlate what exactly the test material was with the nanomaterial under assessment [45]. This in turn makes it difficult to determine if the same nanomaterial was tested across a battery of assays where datasets are provided on different genotoxicity endpoints. The SCCS’s Opinion on Hydroxyapatite (Nano), has noted that a regulatory dossier has provided a good example of the level of physicochemical characterization required for risk assessment purposes [9]. In addition to robust nanomaterial characterization, it is also important to understand if the test nanomaterial reaches the target cell/tissue; again, this information is often not provided. This can be technically challenging to measure for several reasons; e.g. whilst TEM is considered the gold standard for evaluating nanomaterial uptake, it cannot be applied to all materials (e.g. carbon-based materials). Also, TEM is not always quantitative, and it is a highly time-consuming technique, which is not amenable to high-throughput approaches. Other methods exist that can be more quantitative, such as flow cytometry-based analysis and inductively coupled mass spectrometry (ICP-MS), but these techniques do not discriminate between material attached to the surface of the cell versus those particles that are internalized. Nonetheless, evaluating cell/tissue internalization is essential supporting information to justify the need on whether to follow up on *in vitro* results with further *in vivo* testing, particularly for those study outcomes that are negative for genotoxicity. In the case of the *in vivo* genotoxicity assays, it is also necessary given the concerns of whether nanomaterials can reach the systemic tissues (e.g. bone marrow), which is essential if the output from these genotoxicity tests are to be relied upon. In the absence of evidence to demonstrate the test nanomaterial had interacted with the target cells, the assay results could be considered inconclusive. Indeed, due to the low bioavailability of most nanomaterials, irrespective of the route of exposure, the detection of low amounts of nanomaterials at the systemic level can be challenging following a short-term exposure but is more readily ascertained with a repeated exposure scenario, due to potential bioaccumulation in key organs. Thus, there is a growing need for further clarification and detailed guidance on the accompanying nanomaterial data (including physicochemical characterization and evaluation of cell/tissue interaction) to supplement the results generated via a standard testing approach. Without this supporting information it is often difficult for regulatory risk assessors to conclude on genotoxicity.

Another deficiency identified is the fact that the scientific literature has limitations in both test methodology and data reporting, which may also be associated with the conflicting reports published on nanomaterial hazard assessment. Uncertainty is usually caused by a lack of reporting on the test substance, how sampling and dispersion were conducted, and in some cases, how the genotoxicity test was performed (including the use of appropriate assay controls, and controls for potential interference). This information is required for regulatory purposes.

Some shortcomings in the standard testing approaches remain for nanomaterials. The issues surrounding the *in vitro* micronucleus assay (OECD TG487) have been improved and a new OECD GD on the adaptation of this assay for testing of manufactured nanomaterials, has been published (Series on Testing & Assessment No. 359; ENV/CBC/MONO(2022)15) [17]. However, concern remains with the relevance of the *in vivo* micronucleus assay, given the limited likelihood of nanomaterials reaching the bone marrow. Therefore, it was recommended to consider instead new approaches focussing on organs/tissues whether representative of the site of contact exposure and/or where *in vivo* accumulation is expected (e.g. lung, liver, and spleen). The *in vivo* comet assay OECD 489, which can be done in a broad range of organs/tissues, or the *in vivo* micronucleus assay on liver or stomach and colon epithelium are likely the most appropriate [58, 59]. Indeed, it was concluded by the 7th International Workshop on Genotoxicity Testing (IWGT) that the liver micronucleus test was sufficiently validated for the development of an OECD TG while the GIT micronucleus test gave promising results but required further validation [60]. As previously noted, the short-term dose administration design proposed in the OECD TG 474 and 489 may not be appropriate to investigate the genotoxic effects at the systemic level since most of the nanomaterials have been shown to have a low bioavailability. Therefore, a longer exposure time or coupling the *in vivo* micronucleus and/or comet assays with 28- or 90-day toxicity tests can be more valuable. As EU Cosmetics regulation has prohibited *in vivo* testing, data instead need to come from *in vitro* assays and *in silico* models in place of data from an organism. This approach is only workable when based on WoE, instead of any single test result. When such data from validated or scientifically valid tests and models are combined in a WoE, the regulatory risk assessors have more confidence in making a conclusion even when there may be a deviation from the standard approaches. This may therefore be an important way forward to consider for nanomaterials where current standard testing approaches suffer deficiencies.

3 What is needed from a regulatory point of view to be able to apply NAMs in risk assessment? (e.g. full validation, confidence).

The panel recognized that full validation takes an exceptional amount of time that delays the application of NAMs in the regulatory context. To overcome this issue, it is therefore critical to demonstrate that the methodology presented is scientifically justified and valid [61, 62]. In these cases, NAMs can be considered in a WoE approach in the absence of full (formal) validation. Furthermore, there is a need to develop high-throughput based NAMs to facilitate the rapid evaluation of large numbers of nanomaterials and advanced materials to support future innovation. This is particularly important given the exponentially increasing variety of nanomaterials, advanced materials, and nano-enabled products that are continually being developed and require regulatory oversight for consumer/environmental safety.

Several international initiatives have been undertaken to explore the issues that need to be addressed to promote the use of NAMs for regulatory purposes [61–65]. The importance of testing the scientific validity of NAMs and their regulatory applicability through fit-for-purpose case studies is generally recognized and practiced [66–69]. In this context, the OECD initiative of promoting the development of Integrated Approaches for Testing and Assessment (IATAs) is of considerable relevance. IATA approaches can in fact integrate data from NAMs to enable a conclusion on chemical safety. Presently around 85% of published OECD IATA Case Studies contain information from NAMs, although only one case study is focussed on the assessment of genotoxicity of nanomaterials (ENV/JM/MONO(2018)28).

To stimulate the application of NAMs for nanomaterials, EFSA recently funded a call for proposal (GP/EFSA/MESE/2022/01—NAMS4NANO) for the implementation of the EFSA roadmap for NAMs [65]. The central goal is to develop case studies representing real examples of risk assessments, combining existing information with newly conducted NAM studies covering the nanoscale considerations. In early 2023, ECHA funded a study on ‘Nano-specific alternative methods in human risk/safety assessment under different EU regulations, considering the animal testing bans already in place for cosmetics and its ingredients’ i.e. based on a systematic literature review on the currently available, nano-specific replacement methods for the testing of the safety of nanomaterials (Tender ECHA/2022/62). In 2022, the EU Horizon 2020 project PARC (Partnership for the Assessment of Risks from Chemicals) started, with a wide and innovative partnership that involves 28 European countries, aimed at facilitating the transition to next generation risk assessment (NGRA) in which *ad hoc* testing strategies and validation procedures could facilitate the integration of NAMs into risk assessment [70]. These and other similar initiatives, although not specifically focussed on nanomaterials, will contribute to building scientific confidence in the use of NAMs, paving the way for NGRA implementation.

4. What are your thoughts about the possibility of having a guideline for genotoxicity testing of nanomaterials including recommendations for uptake evaluation and characterization?

To support genotoxicity testing, there is a requirement for additional supporting data to supplement the DNA damage testing reports. This includes the need to have robust physicochemical characterization of the test nanomaterial, including data on both the intrinsic and extrinsic features of the material (i.e. the as-manufactured state, and under experimental conditions, respectively). Additionally, as previously noted, it is important to include suitable evaluation of internalization of the test material by the target cell or tissue under assessment. Currently, the provision of supporting nanomaterial characterization data and evidence of target cell/tissue uptake does not routinely accompany genotoxicity testing datasets in the literature and in the regulatory setting (within submitted safety dossiers). Dosimetry and top dose to be tested are also questions that need to be harmonized. However, for many of these measures, OECD TGs, OECD GDs, and harmonized SOPs are not available. Another complication is that the experimental approaches that are selected to generate these datasets will be dependent upon the nanomaterial and route of exposure; thus, several different experimental approaches may be required.

There are ongoing efforts within the EU for different regulatory agencies to work together on harmonization because they work under separate frameworks and may have access to different data. To minimize this redundancy in effort, there is a desire to move towards a one substance–one assessment approach, but this requires a significant change. For example, improvements in communication are being made between agencies to know whether they are evaluating the same/similar substances, or even have the same data or experts. This new approach will reduce the possibility of different agencies coming up with different assessments when applying WoE considerations. It is therefore crucial to ensure all parties involved are seeing the same (complete) set of data/information, and that there is effective communication among EU agencies to prevent conflicting conclusions on nanomaterial hazards. Furthermore, the provision of overarching guidelines outlining the supporting data i.e. required to supplement genotoxicity testing (such as for physicochemical characterization and evidence for cell/tissue internalization) would aid in facilitating a more transparent and uniform approach to nanomaterial risk assessment.

## Conclusions and outcomes

The main conclusions of the workshop were:

- The genotoxicity assessment of nanomaterials remains challenging, and further development of nano-specific OECD TGs and GDs is required.
- Current deficiencies exist in:
  - Exposure regime—traditional acute dosing is not representative of the true human exposure scenarios; longer-term and/or repeated exposures are required to exclude genotoxicity/carcinogenicity potential of nanomaterials; and the top concentration and concentration range need to be adapted.
  - Sufficient characterization of intrinsic and extrinsic nanomaterial physicochemical features and dosimetry is necessary to correlate the physicochemical aspects of the material as well as its concentration with toxicological responses.
  - Evaluation of target cell/tissue internalization—development of experimental approaches, that are preferably high throughput, are required to confirm and measure the uptake and accumulation of nanomaterials in cells and tissues.
  - Adequate coverage of genotoxic modes of action—novel *in vitro* approaches need to be developed to detect both genotoxic and non-genotoxic carcinogens.
- NAMs can strongly support the genotoxicity assessment of nanomaterials, by providing (additional) insights into the mechanisms of action of the materials. However, in the absence of validation, it is important to at least demonstrate the scientific validity of a NAM and its regulatory applicability through fit-for-purpose case studies (e.g. representing real examples of risk assessments, combining existing information with newly conducted NAM studies).
- There is a growing need for further clarification and detailed guidance, potentially a new supplementary OECD GD, on the accompanying nanomaterial data (including physicochemical characterization, evaluation of cell/tissue interaction, and harmonized data templates) to supplement genotoxicity testing. Without this supporting information it is often difficult for regulatory risk assessors to conclude on the genotoxicity of a nanomaterial.
- A closer engagement between scientists and regulators are needed to: (i) provide clarity on the regulatory needs, (ii) improve the acceptance and use of NAM-generated data, and (iii) define how NAMs may be used in WoE framework for regulatory purposes.
